# The complete mitochondrial genome of *Zamia integrifolia* uncovers distinct patterns of genomic turnover and repeat expansion in Zamiaceae

**DOI:** 10.3389/fpls.2026.1939158

**Published:** 2026-09-22

**Authors:** Zhaowu Zhang, Shanshan Dong, Zhijing Qiu, Yunhua Wang, Ting Chen, Yiqing Gong, Tengbo Huang, Hui Wang

**Affiliations:** 1 Guangdong Provincial Key Laboratory for Plant Epigenetics, College of Life Sciences and Oceanography, Shenzhen University, Shenzhen, China; 2 Key Laboratory of Southern Subtropical Plant Diversity, Fairy Lake Botanical Garden, Shenzhen & Chinese Academy of Sciences, Shenzhen, China

**Keywords:** Cycadales, cyto-nuclear discordance, genomic turnover, mitochondrial genome, repeat expansion, *Zamia integrifolia*

## Abstract

**Introduction:**

Gymnosperm mitochondrial genomes exhibit substantial variation in size and structure, yet the evolutionary dynamics of Cycadales remain insufficiently explored beyond the genus Cycas.

**Methods:**

We assembled the complete mitochondrial genome of Zamia integrifolia using Oxford Nanopore long-read and MGI short-read sequencing. Genome assembly, polishing, and annotation were performed through an integrated bioinformatics pipeline. Repetitive elements and mitochondrial plastid DNA transfers (MTPTs) were characterized. Whole-genome synteny and structural rearrangements were analyzed. Phylogenomic relationships and topological discordance (gene and site concordance factors, gCF/sCF) were evaluated with gene. Lineage-specific substitution rates (*dN*, *dS*, and *ω*) were estimated under a branch-specific model.

**Results:**

The genome (487,460 bp) reveals a ~74 kb expansion relative to Cycas, driven primarily by the accumulation of non-redundant repeats (107,836 bp) and extensive plastid DNA integration (63,674 bp). Quantitative metrics indicate a process of active turnover where ancestral spacers are purged and replaced by lineage-specific elements. Despite this structural flux, the gene content and local order remain conserved under purifying selection. Our phylogenetic reconstruction positions Cycadales as the sister group to all other extant gymnosperms, although gene-tree support for this node is low (gCF = 34.8%; sCF = 44.0%),and this topology is discordant with the Cycadales–Ginkgoales clade often recovered in nuclear datasets. We discuss how the exceptionally low substitution rates in cycad mitogenomes may preserve ancestral polymorphisms, leading to incomplete lineage sorting (ILS) that confounds deep node resolution, and we also consider long-branch attraction (LBA) and other sources of systematic error as alternative explanations for the observed discordance.

**Discussion:**

These findings highlight a distinct evolutionary mode in Zamiaceae involving structural fluidity amidst sequence stasis and underscore the complexity of resolving gymnosperm phylogeny using organellar markers.

## 1 Introduction

A feature of plant mitochondrial genomes is the disconnect between the rapid structural evolution of mitochondrial genomes (mitogenomes) and the extremely slow evolution of their coding sequences (Palmer and Herbon, 1988; Putintseva et al., 2020). Unlike animal mitogenomes, which are generally conserved and compact, land plant mitogenomes vary substantially in size from the minimal ~66 kb in some parasites to over 11 Mb in conifers (Skippington et al., 2015; Putintseva et al., 2020). Understanding the mechanisms underlying this variation is important for elucidating organellar genome evolution. Current models suggest three main drivers for mitogenome expansion: (1) the proliferation of repetitive DNA, especially large repeats that trigger homologous recombination (HR) and structural rearranging (Liu et al., 2022a; Wang et al., 2024a; Kong et al., 2025); (2) the capture of foreign DNA via intracellular gene transfer (IGT), including plastid derived sequences (Mitochondrial Plastid DNA, MTPT) (Wang et al., 2007; Zhao et al., 2019; Kong et al., 2025); and (3) the accumulation of non-coding DNA in intergenic spacers due to relaxed selective pressure under low mutation rates (Wang et al., 2024b).

Recent literature emphasizes the mutation hazard hypothesis (MHH) and the role of repair pathways—such as HR versus non-homologous end joining, in deciding the fate of non-coding DNA (Wang et al., 2024b). For example, when homologous templates are scarce, substitution rates may spike; conversely, efficient HR repair tends to preserve sequence fidelity but can inadvertently promote structural isomerism and repeat expansion (Wang et al., 2024a, b). Because complex, repeat-heavy mitogenomes are known to be difficult to assemble, our knowledge has been skewed toward angiosperms, leaving early-diverging seed plants (gymnosperms) under-studied (Wang et al., 2024a).

Gymnosperms span a wide range of mitogenomic variation (Guo et al., 2016). On one end, lineages like the Pinaceae harbor multi-megabase genomes expanded with repeats and foreign DNA (Putintseva et al., 2020); on the other, Cycas (Cycadaceae) maintains an unusually compact and static structure (Chaw et al., 2008; Habib et al., 2021). This contrast prompts examination of whether the mitogenomic stasis observed in *Cycas* is representative of Cycadales as a whole. Is the stasis seen in *C. debaoensis* the ancestral norm for all cycads, or does the sister family, Zamiaceae, engage in the dynamic expansion seen elsewhere in gymnosperms? To answer this, we assembled the complete *Z. integrifolia* mitogenome using Nanopore sequencing, which facilitates the resolution of repeat mediated genomic structures (Kong et al., 2025; Tang et al., 2025). We further examined the phylogenetic placement of Cycadales and the evolution of mitogenomic substitution rates across gymnosperms, using gene and site concordance factors (gCF/sCF) and lineage-specific *dN*, *dS*, and *ω* estimates, to assess how sequence-level processes relate to the structural dynamics reported here.Our comparison with *Cycas* and other gymnosperms revises the idea of universal stasis. We found that *Z. integrifolia* has undergone a unique form of genomic turnover, expanding by ~74 kb relative to *Cycas*. This expansion is primarily driven by lineage specific repeat proliferation and plastid DNA integration, which actively replace ancestral intergenic spacers rather than reflecting stochastic drift.

## 2 Results

### 2.1 Genome organization and comparison with gymnosperm lineages

The mitochondrial genome of *Z. integrifolia* was assembled into a single circular molecule with a total length of 487,460 bp, exhibiting a typical AT-rich nucleotide composition with a GC content of 46.25% (**Figure 1**). Phylogenetically, the genome size of *Z. integrifolia* occupies an intermediate position among gymnosperms. Compared with the compact genomes of Cycadaceae (e.g., *Cycas* debaoensis (Habib et al., 2021), *Cycas* taitungensis (Chaw et al., 2008), ~414 kb) and the basal gymnosperm *Ginkgo biloba* (Guo et al., 2016) (346 kb), it exhibits moderate expansion (approximately 74 kb larger). However, it remains significantly smaller than the extremely expanded genomes of *Welwitschia mirabilis* (Guo et al., 2016) (978 kb) and members of the Pinaceae [ranging from 1.19 Mb in Pinus taeda (NC_039746.1) to 11.7 Mb in *Larix sibirica* (Putintseva et al., 2020)].

**Figure 1:**
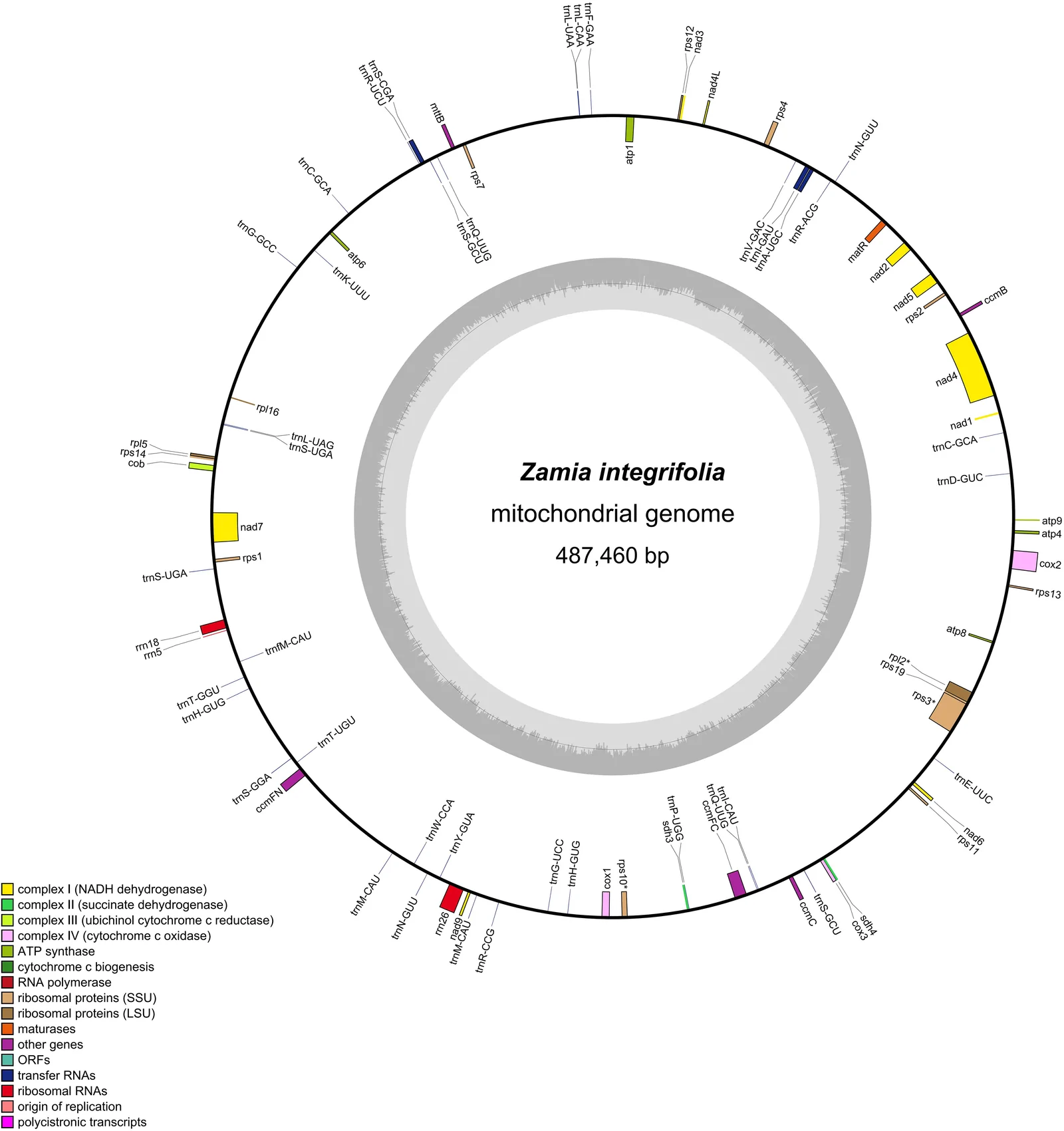
Circular map of the *Z. integrifolia* mitochondrial genome. Genes drawn on the outside of the circle are transcribed in the clockwise direction, while those on the inside are transcribed in the counter-clockwise direction. The functional classification of genes is color-coded according to the legend at the bottom left. The inner dark gray circle represents the GC content plotted using a sliding window. Genes containing introns are marked with an asterisk (*).

The *Z. integrifolia* mitogenome mirrors the AT-rich nature of *C. taitungensis* (~46.9%) (Chaw et al., 2008; Habib et al., 2021) and aligns with the <50% range typical of lycophytes and basal angiosperms (Tang et al., 2023). This profile stands in marked contrast to the high-GC (>50%) architectures seen in *G. biloba*, *Welwitschia mirabilis* (Guo et al., 2016), and most ferns (Knoop, 2013; Guo et al., 2017; Feng and Wicke, 2022; Huang et al., 2025).

While genome size fluctuates, gene content is largely conserved. Our annotation identified 80 unique genes within the assembly, comprising 40 protein-coding genes (PCGs), 37 tRNAs, and 3 rRNAs (rrn5, rrn16, rrn26) (**Supplementary Table S1**). This genome retains the complete set of succinate dehydrogenase genes (*sdh3* and *sdh4*). The total length of protein-coding sequences encodes 11,345 amino acids, accounting for only 6.98% of the total genome length.

### 2.2 Substantial expansion and compartmentalization of repeats

Unlike the comparatively static repeat profile of *Cycas*, the *Z. integrifolia* mitogenome is defined by a substantial buildup of repetitive sequences. Using a non-redundant merging approach to remove overlapping coordinates, we determined the total physical length of Repeats of Unusual Size (ROUS) to be 107,836 bp (**Supplementary Table S2**), representing 22.12% of the genome. This repeat load is a significant increase, roughly double the amount found in *C. debaoensis* (~51.7 kb, 12.5%).

These repeats show a distinct bias in distribution. The vast majority (97,302 bp, or 90.2% of total repeats) are found exclusively within intergenic spacers. We found no ROUS overlap within intron regions, which contrasts with *C. debaoensis* mitogenomes where Bpu-like elements often insert into and expand introns. A small portion (~10.5 kb) overlaps with coding regions, but these instances are largely linked to pseudogenized gene fragments or multicopy rRNA genes, leaving core protein-coding sequences intact.

Short ROUS families dominate the repeat. Our data shows that 3,231 repeat units range from 24–49 bp, making up 58.48% of the total count. We detected a lineage specific proliferation of a 24 bp motif, occurring 221 times (**Supplementary Table S2**). No copies were detected in the *C. taitungensis* or *C. debaoensis* mitogenomes, and a search against Dfam returned no significant similarity to known transposable-element families.

### 2.3 Tandem repeats and mechanism of expansion

We also analyzed repeat structures using Tandem Repeats Finder (TRF), identifying 30,674 bp of non-redundant tandem repeats (TRs) (**Supplementary Table S3**), which cover 6.29% of the genome. Although this is a moderate increase over *C. debaoensis* (~23 kb), TR content alone does not explain the full extent of genome expansion.

Overlap analysis showed that 27,133 bp (88.46%) of these TRs are subsets of the identified ROUS. However, TRs make up only about 28% of the total repetitive content (107,836 bp).

### 2.4 Divergence in microsatellite (SSR) dynamics

To explore micro-evolutionary drivers, we compared Simple Sequence Repeats (SSRs) between *Z. integrifolia* and *C. taitungensis* (**Supplementary Table S4**). The analysis uncovered a shift in repeat dynamics: despite *Z. integrifolia* having a larger genome (~487 kb), it contains fewer standard SSRs (44) than *C. taitungensis* (59, ~415 kb). Of the 59 SSRs detected in *C. taitungensis*, 47 were mononucleotide repeats. Among these 47 mononucleotide SSRs, 42 (89.4%) were Poly-C/G types, reflecting a stable, GC-biased background; the remaining 12 SSRs were di- or trinucleotide repeats. Conversely, *Z. integrifolia* shows a shift toward AT-rich motifs and greater structural complexity. While *C. taitungensis* lacks SSR motifs larger than trinucleotides, *Z. integrifolia* contains unique high-copy pentanucleotide (e.g., (ACCTA)26, 130 bp) and tetranucleotide arrays (e.g., (TATG)24, 96 bp). Because this comparison is based on a single mitogenome per lineage, the observed SSR differences should be regarded as descriptive and require broader sampling before they can be generalized to Zamiaceae or Cycadales.

### 2.5 Extensive integration of plastid-derived sequences (MTPTs)

Beyond endogenous repeats, we examined exogenous DNA contributions from the plastid genome (MTPTs). To avoid false positives, we strictly filtered out native homologous regions (e.g., *rps4*, *rpl16*, *atpA*) before mapping the remaining plastid-derived fragments.

We identified 63,674 bp of non-redundant MTPT sequences (**Supplementary Table S5**), comprising 13.06% of the total mitochondrial genome. This is nearly a four-fold increase over the 3.8% (~16 kb) seen in *C. debaoensis*. The overlap between MTPTs and ROUS was negligible (only 901 bp).

Additionally, *Z. integrifolia* retains exceptionally large plastid segments. These clusters include almost complete functional units, such as major photosynthetic gene sets (e.g., *psbB*-*psbT*-*psbN*-*psbH*-*petB*-*petD*-*rpoA*-*rps11*) and the plastid rRNA operon. The presence of these diagnostic plastid specific genes within the mitogenome supports the exogenous origin and significant scale of these integrations.

### 2.6 Genome structural evolution

We compared the mitogenomes of *Z. integrifolia*, *C. taitungensis*, and *G. biloba* using Mauve (**Figure 2**). The analysis revealed a dynamic landscape of subgenomic evolution. The three genomes share a set of Locally Collinear Blocks (LCBs), but their positions and orientations are extensively shuffled. Many crossing lines between the target and reference genomes indicate frequent translocations and inversions.

**Figure 2:**
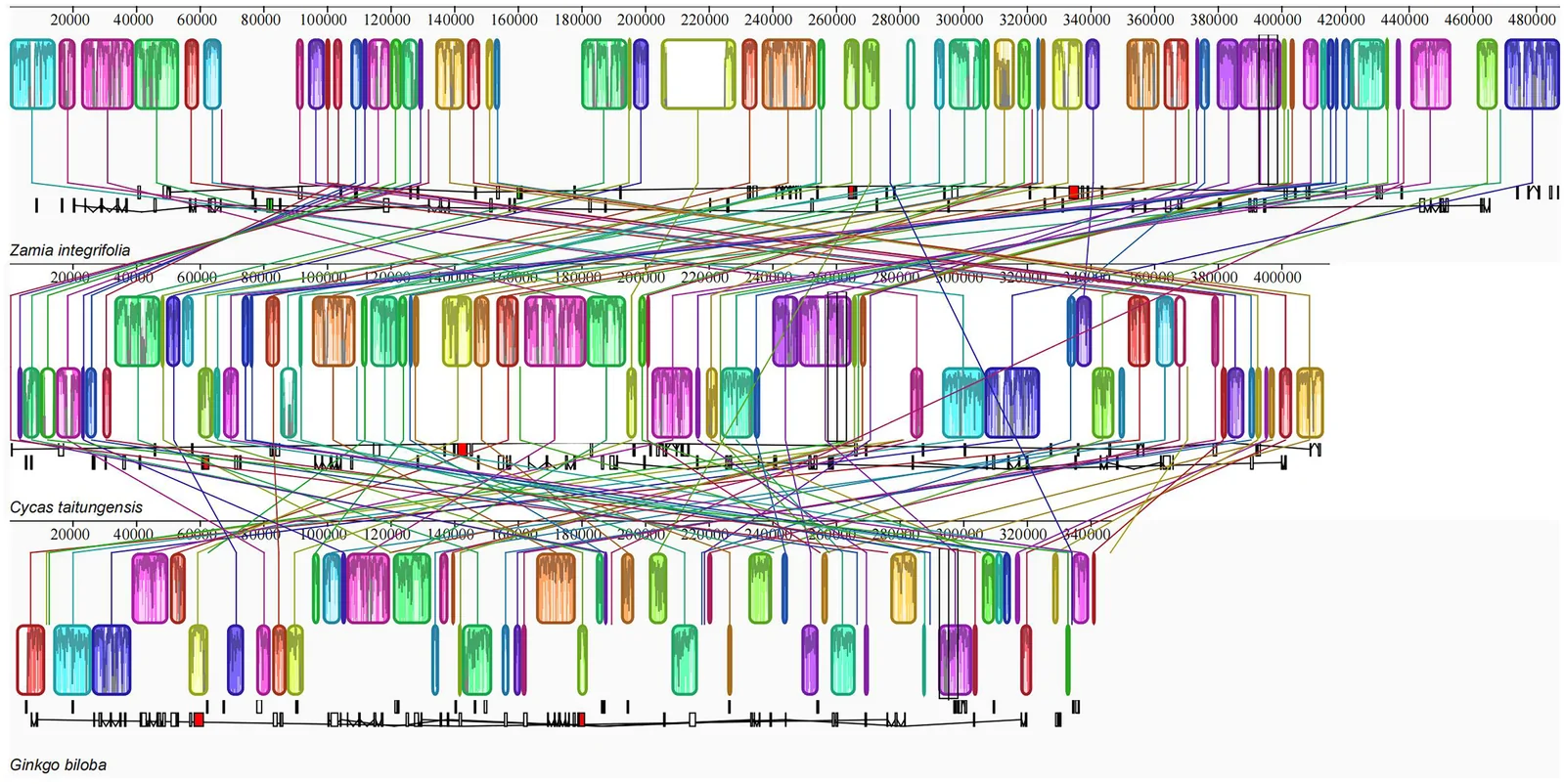
Whole-genome synteny alignment of *Z. integrifolia* with *C. taitungensis* and *G. biloba* using Mauve. The genomes are aligned from top to bottom: *Z. integrifolia*, *C. taitungensis*, and *G. biloba*. Colored blocks represent LCBs, indicating homologous regions without internal rearrangements. Lines connect corresponding LCBs across the genomes. Blocks drawn above the center line are in the forward orientation, while those below are inverted. The vertical height of the colored bars within blocks represents the level of sequence conservation.

We identified 260,305 bp of sequence shared between *Z. integrifolia* and *C. taitungensis*, representing 53.4% of the *Z. integrifolia* mitogenome and 62.7% of the *C. taitungensis* mitogenome (**Supplementary Figure S3**). Within these dispersed but conserved blocks, the functional gene repertoire and local gene order remain stable.

Outside this pairwise core, *C. taitungensis* contains approximately 154.6 kb of sequence, whereas *Z. integrifolia* contains approximately 227.2 kb. Of the *Z. integrifolia* non-core sequence, approximately 171 kb represents newly acquired DNA, consisting of integrated plastid fragments (~63 kb MTPTs) and lineage-specific repeats (107.8 kb ROUS), leaving approximately 56 kb of other non-core sequence. Thus, the two cycad genomes differ mainly in the amount of newly acquired sequence, rather than in a simple loss of ancestral sequence from *Z. integrifolia*.

To assess the direction of this difference, we used *G. biloba* as an outgroup. Sequence shared by *G. biloba* and C. taitungensis but absent from *Z. integrifolia* totaled 6,676 bp, which we interpret as lineage-specific loss in *Z. integrifolia*, whereas 125,475 bp were unique to *Z. integrifolia* and absent from both reference genomes, representing lineage-specific acquisition. Relative to *G. biloba*, the *Z. integrifolia* block order required 120 breakpoints and involved 33 block-orientation inversions among 251 syntenic blocks (**Supplementary Table S6**).

Despite extensive genomic map shuffling, the functional integrity of core genes is preserved. Absolute substitution rates in coding sequences also remain very low. These findings indicate that sequence conservation coexists with structural fluidity in the *Z. integrifolia* mitogenome.

### 2.7 C-to-U RNA editing in the *Z. integrifolia* mitogenome

C-to-U RNA editing was systematically mapped across the protein-coding genes of the *Z. integrifolia* mitogenome. A total of 173 high-confidence editing sites were identified (**Supplementary Table S7**). The distribution of editing events was uneven among loci (**Figure 3**).

**Figure 3:**
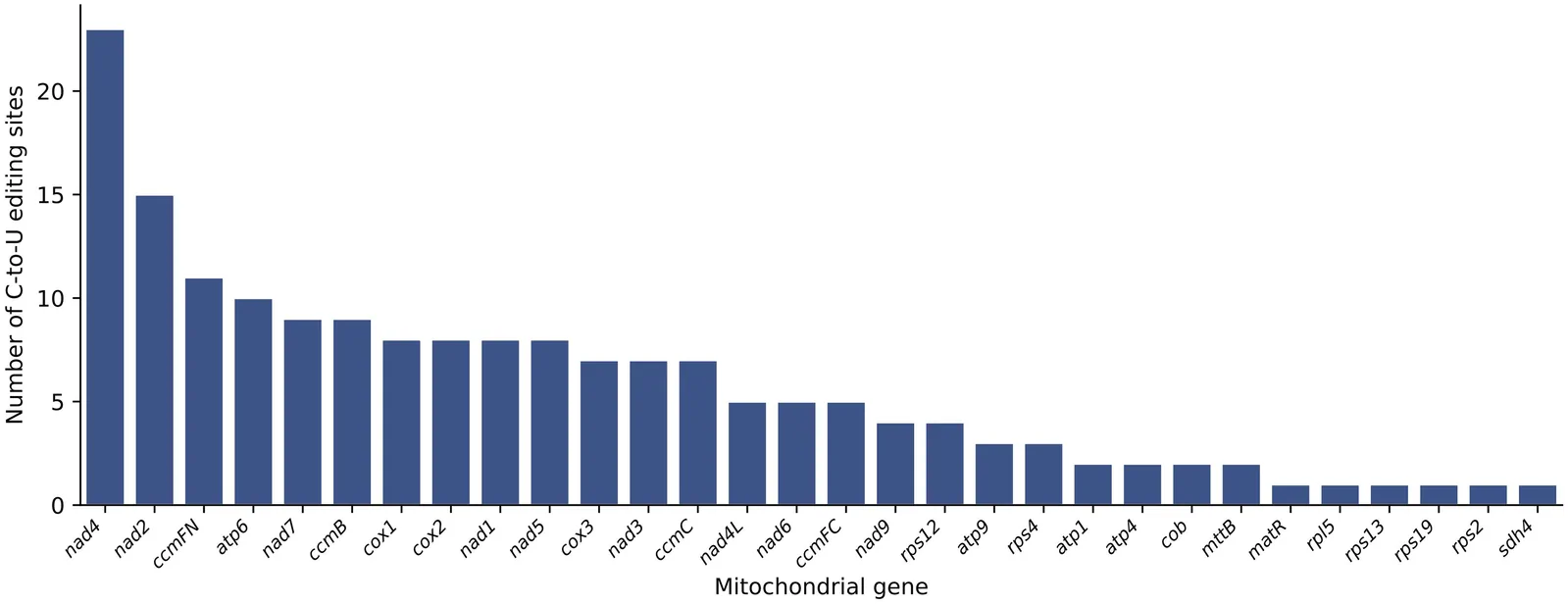
Number of C-to-U RNA editing sites identified in each mitochondrial protein-coding gene of *Z. integrifolia*. Genes are ordered by decreasing number of editing sites.

Editing sites were predominantly concentrated in genes encoding subunits of the respiratory chain and cytochrome c maturation complexes. Among the nad gene family (Complex I), *nad4* (23 sites) and *nad2* (15 sites) had the most edits, followed by *nad7* (9), *nad1* (8), and *nad5* (8). Genes involved in cytochrome c biogenesis also harbored numerous edits, notably *ccmFN* (11), *ccmB* (9), and *ccmC* (7). High editing activity was further observed in *atp6* (10), *cox1* (8), and *cox2* (8).

Analysis of the edited nucleotide coordinates revealed a bias toward the second and first codon positions (122 and 50 sites, respectively, together 99.4% of all sites), whereas third-position edits were extremely rare (1 site). Consequently, 171 of 173 edits (98.8%) were non-synonymous. The most frequent amino acid conversions were Serine-to-Leucine (52), Proline-to-Leucine (38), Serine-to-Phenylalanine (25), and Arginine-to-Cysteine (17). These substitutions generally increase residue hydrophobicity and are expected to stabilize the transmembrane helices of membrane-associated respiratory proteins.

RNA editing in the *Z. integrifolia* mitogenome serves is a prevalent post-transcriptional modification that mainly restores conserved amino acid residues and may help maintain the functional integrity of respiratory proteins.

### 2.8 Synonymous and non-synonymous substitution rates

To further evaluate the evolutionary history and selective pressures acting on mitochondrial genes, we estimated lineage-specific synonymous (*dS*) and non-synonymous (*dN*) substitution rates for 35 mitochondrial protein-coding genes using the free-ratio branch model implemented in PAML, after correcting the *Z. integrifolia* CDS for the 173 C-to-U RNA editing sites (**Supplementary Table S8**). Across the three lineages (*Z. integrifolia*, *C. taitungensis*, and *G. biloba*), both synonymous and non-synonymous substitution rates remained generally low, consistent with the exceptionally slow evolutionary dynamics of plant mitochondrial genomes (**Figure 4**). Branch lengths were relatively short for most genes in both cycad lineages, indicating limited sequence divergence following the split of Cycadaceae and Zamiaceae, whereas the *G. biloba* lineage generally exhibited greater synonymous divergence.

**Figure 4:**
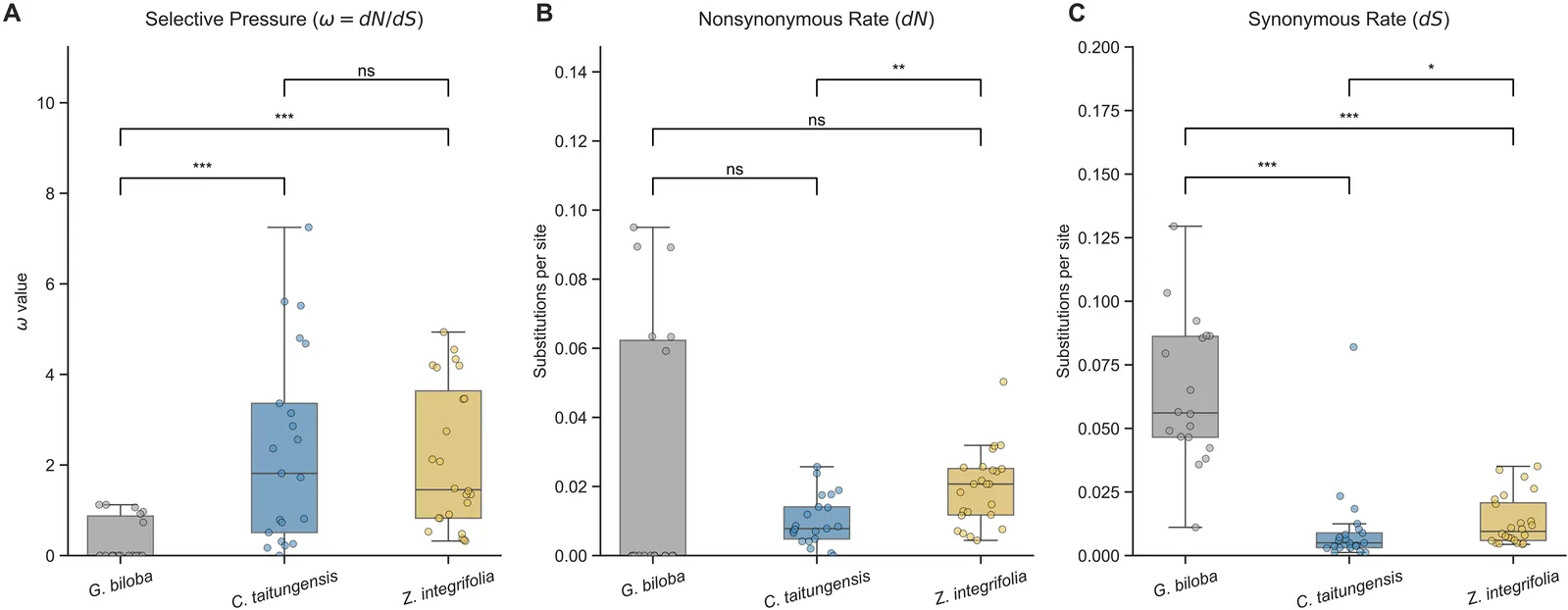
Evolutionary rates and selective pressure dynamics of mitochondrial genes across *G. biloba*, *C. taitungensis*, and *Z. integrifolia*. Boxplots illustrate the distribution of **(A)** selective pressure (ω=*dN/dS*), **(B)** non-synonymous substitution rate (*dN*), and **(C)** synonymous substitution rate (*dS*) estimated from conserved mitochondrial genes across the three lineages. Colors distinguish the specific lineages: *G. biloba* (grey), *C. taitungensis* (blue), and *Z. integrifolia* (yellow). Individual genes are represented as jittered dots overlaid on the boxplots. Within each box, the central horizontal line marks the median, while the upper and lower bounds indicate the interquartile range (IQR). Statistical significance between pairs was evaluated using the two-sided Mann-Whitney U test followed by Bonferroni correction for multiple comparisons (ns, not significant; *P < 0.05; **P < 0.01; ***P < 0.001). Extreme artifactual values resulting from mutational saturation were excluded for robustness.

Gene by gene analyses uncovered heterogeneous evolutionary patterns among mitochondrial genes. The median ω was 1.064 in *G. biloba*, 4.681 in *C. taitungensis*, and 3.455 in *Z. integrifolia*. Nevertheless, several genes, including *atp1*, *atp6*, *ccmFN*, *cob*, *cox1*, *cox2*, *nad2*, *nad4*, *rps4*, and *rps7* in the *C. taitungensis* lineage, and *atp4*, *atp6*, *ccmB*, *ccmC*, *cox2*, *cox3*, *nad1*, *nad2*, *nad4*, *nad4L*, and *nad6* in the *Z. integrifolia* lineage, displayed relatively elevated ω values (>2). Inspection of the underlying substitution rates revealed that these elevated ω estimates were generally associated with extremely low synonymous substitution rates (dS ≤ 0.005), indicating that the large ω values largely resulted from limited synonymous substitutions rather than substantial increases in non-synonymous evolution.

Including *atp8*, *atp9*, *ccmB*, *cob*, *cox3*, *mttB*, *nad1*, *nad3*, *nad4L*, *nad6*, *rpl5*, *rps11*, *rps12*, *rps13*, *rps14*, *rps1*, *rps2*, and *sdh3*, reached the upper boundary estimate (ω = 999) in one or more lineages. In nearly all of these cases, *dS* approached zero, reflecting mathematical inflation of ω caused by the absence of synonymous substitutions rather than robust evidence for positive selection. Similar patterns have frequently been reported in slowly evolving plant mitochondrial genomes, where extremely low synonymous substitution rates can produce unstable ω estimates.

Although *G. biloba* generally exhibited longer branch lengths and higher synonymous divergence than the two cycad lineages, non-synonymous substitution rates remained comparatively low for most genes. Notably, rpl16 in *Z. integrifolia* displayed the highest absolute substitution rates (*dN* = 0.8355, *dS* = 2.7886) while maintaining a low ω value (0.30), indicating accelerated sequence evolution accompanied by proportionally elevated synonymous substitutions and continued functional constraint. Likewise, genes such as *rps4* and *ccmFN* retained ω values close to or below one in *G. biloba*.

These results indicate that mitochondrial protein-coding genes remain conserved among the three gymnosperm lineages examined. Although elevated ω values were observed in several lineage-specific genes, these estimates were primarily driven by extremely low synonymous substitution rates rather than providing convincing evidence for lineage-specific positive selection, highlighting the strong evolutionary constraints acting on mitochondrial coding sequences.

### 2.9 Codon usage bias and compositional features

We analyzed nucleotide composition and codon usage for the 40 PCGs in *Z. integrifolia*. There is a clear bias toward Adenine (A) and Thymine (T). Base frequencies in coding regions were 27.8% (T), 23.2% (C), 26.6% (A), and 22.4% (G), yielding a GC content of 45.6%. GC content declines progressively from the first (GC_1_: 49.4%) and second (GC_2_: 47.2%) to the third position (GC_3_: 40.1%). The high AT-enrichment at the third position (59.9%) suggests that synonymous sites face strong mutational pressure toward A/T.

RSCU analysis of 11,348 codons identified 30 preferred codons (RSCU > 1.0) (**Figure 5**). Remarkably, 29 of these (96.7%) end in A or U, confirming that AT-biased mutational pressure drives codon usage. The most preferred codons were GCU (Ala), UAU (Tyr), CAU (His), and CAA (Gln). In contrast, C/G-ending codons like UAC and GGC were underrepresented. Among termination codons, UGA (1.20) was the most frequent, followed by UAG and UAA. Consistent with land plant trends, Leucine and Serine were the most abundant amino acids, dominated by their A/U-ending codons.

**Figure 5:**
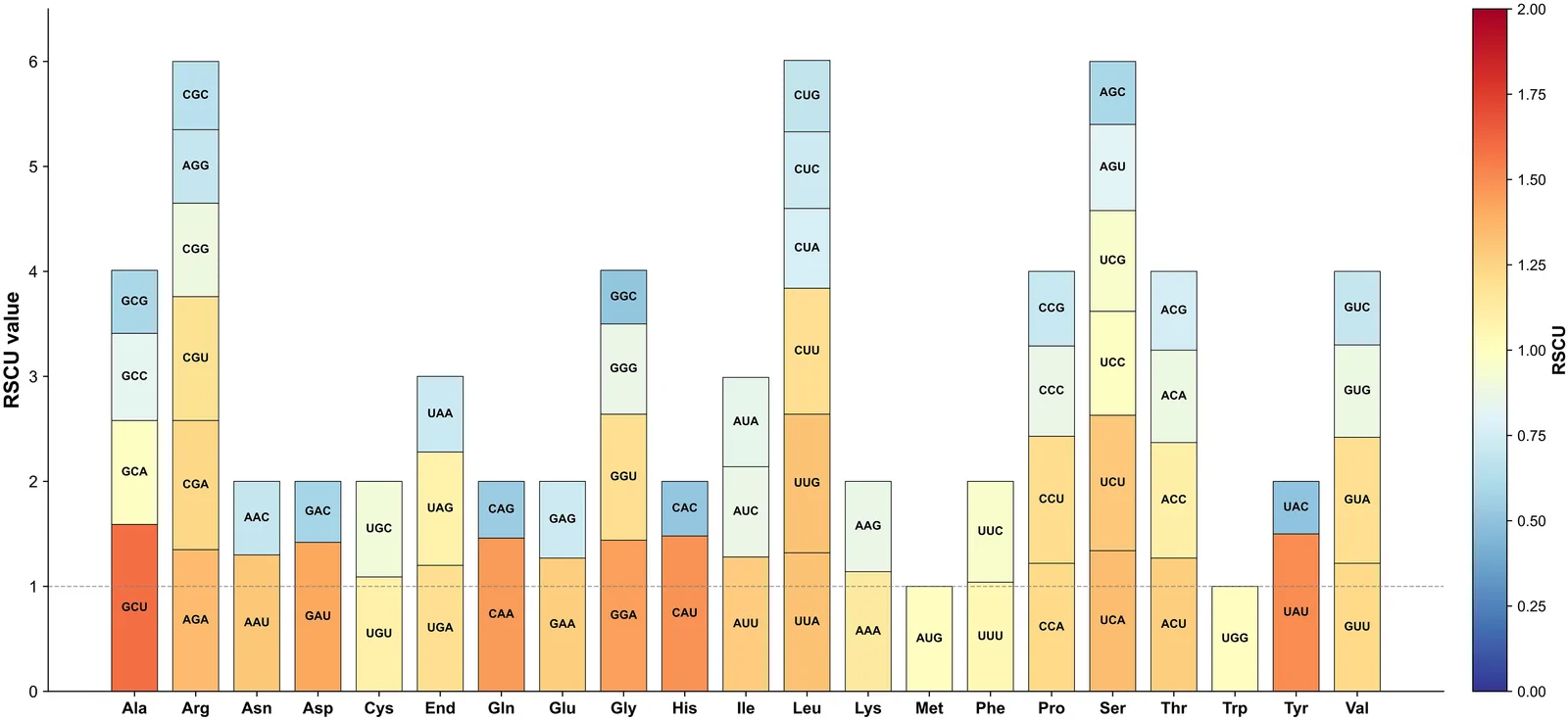
RSCU patterns within the *Z. integrifolia* mitochondrial coding regions. The X-axis categorizes amino acids alongside termination codons. Stacked bars depict the total codon volume per amino acid, with color-coded subdivisions illustrating the RSCU metric for individual codons. We classified codons as preferred if their RSCU exceeded 1.0, and underrepresented if the value fell below 1.0.

### 2.10 Phylogenetic relationships and mitochondrial gene content evolution

To investigate the evolutionary history of gymnosperm mitogenomes, we constructed a Maximum Likelihood (ML) tree from an amino-acid of 26 conserved mitochondrial protein-coding genes across 13 taxa, including 12 gymnosperm ingroups and the angiosperm Amborella trichopoda as outgroup. Best-fit substitution models were independently assigned to each gene partition to account for rate heterogeneity (**Supplementary Table S9**). In the resulting phylogeny (**Figure 6**), after rooting at Amborella, *Z. integrifolia* and *C. taitungensis* formed a maximally supported clade (UFBoot = 100; gCF = 95.7%; sCF = 85.1%; ASTRAL support = 1.00; **Supplementary Table S10**). This sister relationship was supported by high gene and site concordance factors. We also observed heterogeneity in branch lengths across the tree; for instance, the branch leading to Welwitschia (Gnetales) was relatively long (branch length ≈ 0.316), whereas the Cycadales branches were shorter (*C. taitungensis* ≈ 0.013; *Z. integrifolia* ≈ 0.034).

**Figure 6:**
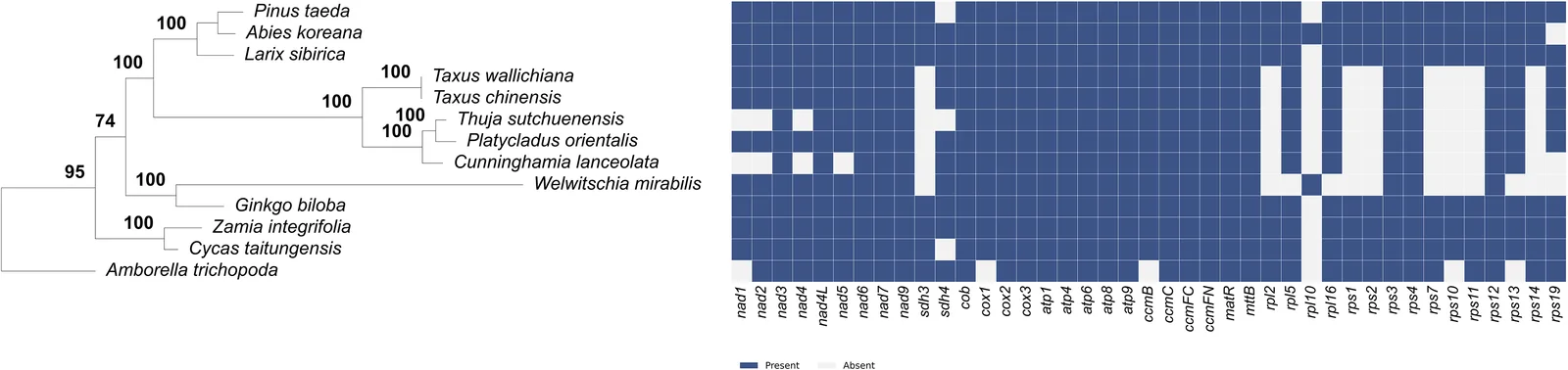
Maximum likelihood (ML) phylogenetic tree and mitochondrial gene content evolution across major gymnosperm lineages. (Left) The phylogenetic tree was reconstructed using the amino acid sequences of 26 conserved mitochondrial PCGs with IQ-TREE. Amborella trichopoda serves as the outgroup. Numbers at the nodes indicate ultrafast bootstrap support values (%). (Right) The heatmap illustrates the presence (blue) and absence (white) of 41 mitochondrial PCGs across the sampled species. Functional genes are grouped by complex (e.g., atp, ccm, nad) and ribosomal proteins (rpl, rps).

In the concatenated ML tree, Cycadales was recovered as the sister group to all other extant gymnosperms, and Ginkgo formed a clade with Welwitschia. After rooting at Amborella, the clade comprising all non-cycad gymnosperms (including Ginkgo, Welwitschia, and conifers) received UFBoot = 95%, but only 34.8% of gene trees (8/23) and 44.0% of sites were concordant with this branch. Within this clade, the Ginkgo-Welwitschia clade received UFBoot = 100% but gCF = 56.5% and sCF = 48.4%, whereas the conifer clade (Pinaceae + Cupressophytes) received UFBoot = 74%, gCF = 26.9% (7/26), and sCF = 33.7%. ASTRAL recovered the Cycadales-basal placement with high support (0.97) but placed Ginkgo with conifers and Welwitschia with Cupressophytes (0.75).

To assess the influence of the long Welwitschia branch, we repeated all analyses after excluding Welwitschia. UFBoot, gCF, and ASTRAL support for the non-cycad gymnosperm clade increased (UFBoot: 95% to 98%; gCF: 34.8% to 40.0%; ASTRAL: 0.97 to 1.00), whereas sCF remained comparable (44.0% to 41.1%). The conifer clade became strongly supported (UFBoot: 74% to 100%; gCF: 26.9% to 50.0%; sCF: 33.7% to 40.8%). After exclusion, Ginkgo no longer clustered with Welwitschia; instead, it formed a separate lineage sister to the conifer clade in both the ML and ASTRAL analyses.

The conserved nature of cycad mitogenomes is also reflected in their mitochondrial gene repertoire. Comparative analysis of gene presence and absence (**Figure 6**) indicated that *Z. integrifolia* and *C. taitungensis* share a similar and relatively complete ancestral gene set. While core functional genes are largely conserved across the surveyed gymnosperms, the Cycadales lineages have retained a broader suite of ribosomal protein genes (rpl and rps families) and succinate dehydrogenase subunits (*sdh3* and *sdh4*).

## 3 Discussion

The mitochondrial genome of Cycas is frequently cited as an example of evolutionary stasis (Salmans et al., 2010; Habib et al., 2021). However, comparison with *Z. integrifolia* suggests that this stability is not conserved across Cycadales. *Z. integrifolia* exhibits pronounced intergenic turnover. Although the net increase in genome size (~74 kb) is modest relative to the large expansions reported in Pinaceae or Welwitschia (Liu et al., 2022b), this net change reflects substantial genomic flux: in the outgroup-polarized Mauve comparison, only 6,676 bp of sequence shared by *G. biloba* and *C. taitungensis* was absent from *Z. integrifolia*, whereas the *Z. integrifolia* mitogenome accumulated 107.8 kb of ROUS and ~63 kb of MTPTs. Because coding capacity is similar to that of *G. biloba* and *Cycas*, the expansion is attributable mainly to elongation of non-coding intergenic regions and accumulation of repetitive sequences. Thus, despite strong conservation of the genic core, non-coding regions in Zamiaceae have experienced marked structural divergence.

Genome expansion in *Z. integrifolia* is associated with the accumulation of endogenous repeats and plastid-derived DNA (MTPTs). In contrast to the GC-rich microsatellites characteristic of Cycas (Chaw et al., 2008), *Z. integrifolia* harbors more complex, AT-rich repeat motifs and a lineage-specific 24 bp motif (ROUS_5513, consensus 5’-CATAGCATGCTATTCTCTAGAGAT-3’, 221 copies) that is absent from the *C. taitungensis* and *C. debaoensis* mitogenomes and shows no significant similarity to known transposable elements. These dispersed repeats and tandem arrays are consistent with amplification by replication slippage rather than transposable-element activity. In addition, plastid-derived sequences comprise 13.06% of the mitochondrial genome and include large clusters that retain nearly complete plastid gene sets; however, they are not annotated as functional mitochondrial genes and are interpreted as non-functional sequence relics. The negligible overlap between MTPTs and ROUS supports that repeat expansion and plastid DNA integration are largely independent contributors to mitogenome expansion. These findings are consistent with the MHH, under which the exceptionally low mutation rates of gymnosperm mitogenomes reduce the selective cost of maintaining excess non-coding DNA (Sudianto et al., 2020; Feng and Wicke, 2025). Under such conditions, repetitive elements and large MTPTs may persist as largely neutral sequence components (Richardson et al., 2013; Wang et al., 2024a, b).

Mitochondrial phylogenetic analyses place Cycadales as the sister group to all other extant gymnosperms, a topology that contrasts with the Cycadales–Ginkgoales clade commonly recovered from nuclear genomic data (Wu et al., 2013; Liu et al., 2018; Offer et al., 2023). This cyto-nuclear discordance is widely observed in gymnosperms and is often attributed to ILS during the rapid early diversification of seed plants (Liu et al., 2022b; Lin et al., 2025). In cycads, the extremely low substitution rates of mitochondrial genes may permit ancestral polymorphisms to persist for extended evolutionary intervals, thereby limiting the resolution of deep phylogenetic nodes. However, the Cycadales-basal branch received UFBoot = 95%, gCF = 34.8%, sCF = 44.0%, and ASTRAL support of 0.97, whereas the conifer clade received UFBoot = 74%, gCF = 26.9%, and sCF = 33.7%. These values indicate substantial gene-tree and site-level discordance; high bootstrap support in the concatenated analysis alone does not demonstrate that the topology is supported by individual genes. The sensitivity analysis excluding Welwitschia increased support for the non-cycad gymnosperm clade and changed the placement of Ginkgo from clustering with Welwitschia to forming a separate lineage sister to the conifer clade. These results suggest that the long Welwitschia branch contributed to deep-node discordance, although appreciable gene-tree conflict persisted at the Cycadales-basal branch after its exclusion. We therefore cannot attribute the observed discordance solely to ILS; it may reflect ILS, LBA, or other systematic artifacts. Relying solely on concatenated mitochondrial supermatrices without quantifying gene-tree and site concordance may overestimate confidence in deep relationships.

The *Z. integrifolia* mitogenome illustrates a striking contrast between high sequence conservation and pronounced structural plasticity. Several mitochondrial genes exhibited extremely low or zero synonymous substitutions, and the elevated ω (*dN/dS*) values observed in genes such as *atp8* or *cox2* are interpreted as numerical artifacts arising from low synonymous substitution rates rather than evidence for positive selection (Buschiazzo et al., 2012). This pervasive sequence stasis suggests the action of highly efficient recombination-based DNA repair processes, potentially including RecA-dependent HR (Rowan et al., 2010). While such mechanisms likely contribute to the long-term preservation of essential coding sequences under purifying selection, their activity on dispersed repetitive elements may inadvertently promote structural rearrangements. Thus, mitochondrial evolution in Zamia represents a distinct mode, functional genes are rigidly conserved, while the intergenic space undergoes continuous turnover.

## 4 Conclusion

We assembled the complete mitochondrial genome of *Z. integrifolia*. This sequence provides an important reference for the Zamiaceae family. Our analysis shows that Cycadales mitogenomes change more than previously thought. We found active sequence turnover in this lineage. The *Z. integrifolia* lineage accumulated large amounts of lineage-specific repetitive and plastid-derived DNA, while loss of ancestral intergenic sequence was limited. At the same time, endogenous TRs expanded rapidly. A large amount of plastid derived DNA also entered the genome.

The expansion of the *Z. integrifolia* mitogenome came from a shift in repeat patterns. The new sequences consist of complex AT-rich motifs. The genome also shows a high tolerance for foreign DNA. These changes occurred mainly in the intergenic regions. The PCGs did not change significantly. These coding sequences remain extremely stable and under purifying selection. These results show that ancient seed plants can maintain a stable functional core while undergoing large-scale structural changes.

## 5 Materials and methods

### 5.1 Plant sampling, DNA extraction and sequencing

The plant tissue of a cultivated *Z. integrifolia* was collected from Shenzhen Fairy Lake Botanical Garden, Shenzhen, China. No specific permission was required for the collection of the plant sample used in the current study. The species was identified by Professor Zhang Shouzhou, and the voucher specimen (No. ZhangSZ2025001) was deposited in SZG (Herbarium of Shenzhen Fairy Lake Botanical Garden, Shenzhen, China). Genomic DNA was isolated using a modified CTAB method (Porebski et al., 1997). The quality and quantity of the extracted DNA were examined using 1% agarose gel electrophoresis and a Qubit 4.0 Fluorometer (Thermo Fisher Scientific, USA), respectively. For long-read sequencing, approximately 20μg of high-quality genomic DNA was subjected to library construction and sequenced on the Oxford Nanopore Technologies (ONT) PromethION platform. Additionally, about 1μg of high-quality DNA was fragmented to construct a paired-end library with an average insert size of 150 bp according to the manufacturer’s instructions, and then sequenced on the MGI-SEQ (Korostin et al., 2020) platform.

### 5.2 Genome assembly and annotation

The mitochondrial genome of *Z. integrifolia* was assembled using HiMT v1.1.0 (Tang et al., 2025) with default parameters. A total of 4,460,359 Nanopore reads (101.30 Gb) were used, with a mean read length of 22.71 kb and an N50 read length of 50.32 kb. The initial HiMT assembly was 484,890 bp (**Supplementary Figure S1**). To independently validate the assembly, the mitochondrial genome was also assembled from the same Nanopore dataset using PMAT v1.5.3 (Bi et al., 2024) with default parameters. The resulting PMAT assembly was compared with the HiMT assembly using whole-genome alignment with MUMmer4 (Delcher et al., 2003), and the alignment was visualized as a dot plot to assess sequence collinearity and potential structural differences. As the two assemblies showed highly consistent genome sizes, sequence collinearity, and overall genome structure (**Supplementary Figure S2**), the HiMT assembly was retained for subsequent polishing and downstream analyses.

The HiMT assembly was subsequently polished using Pilon v1.23 (Walker et al., 2014) with corresponding short reads to improve sequence accuracy. After polishing, the final mitochondrial genome length was 487,460 bp, 2,570 bp longer than the initial assembly. Annotation of PCGs, ribosomal RNAs (rRNAs), and transfer RNAs (tRNAs) was performed using GeSeq (Tillich et al., 2017) and PMGA (Tang et al., 2025), with reference to published mitochondrial genomes of *C. taitungensis* (Chaw et al., 2008). The Organellar Genome DRAW (OGDRAW) (Greiner et al., 2019) tool was utilized for the visualization of the genome map. Non-standard start/stop codons and potentially misannotated frameshifts were manually curated and refined based on homology with closely related species by Geneious (Kearse et al., 2012).

### 5.3 Genome and repeat analysis

The final genome size and GC content were calculated using custom Perl scripts. To characterize repeat elements, we employed two complementary strategies:

ROUS: These were identified by self BLASTn (Madden, 2013) searches (e-value < 1e-5, minimal identity > 90%, minimal length > 100 bp) of the *Z. integrifolia* mitogenome against itself. Overlapping repeat coordinates were merged using BEDtools (Quinlan and Hall, 2010) to obtain the total physical length and genomic proportion of ROUS.

TRs were identified using Tandem Repeats Finder (Benson, 1999) (TRF) v4.09 with default parameters. Overlaps between TRs and ROUS were quantified using BEDtools (Quinlan and Hall, 2010). SSRs were identified using MISA (Beier et al., 2017) with predefined motif lengths. The number and distribution of SSRs were compared with *C. taitungensis*.

### 5.4 Whole-genome synteny analysis

Whole-genome alignment between *Z. integrifolia* and *C. taitungensis* was performed using MUMmer v4.0.0beta2 (Delcher et al., 2003) to identify conserved syntenic blocks and genome rearrangements. The nucmer and delta-filter modules were used to generate a non-redundant set of alignments, which were then visualized using the mummerplot utility. Synteny was further visualized and analyzed using Mauve (Darling et al., 2004) to identify LCBs.

The direction of sequence turnover was polarized using *G. biloba* as the outgroup: sequence shared by *G. biloba* and *C. taitungensis* but absent from *Z. integrifolia* was classified as lineage-specific loss, whereas sequence unique to *Z. integrifolia* and absent from both reference genomes was classified as lineage-specific acquisition. For pairwise comparisons, syntenic blocks were counted from the Mauve alignment; breakpoints were defined as the number of block-order adjacencies in the second-listed genome that are absent from the first-listed (reference) genome, and inversions were defined as shared blocks whose orientation differs between the two genomes.

Mitochondrial plastid DNA transfers (MTPTs) were identified by BLASTn (Madden, 2013) searches of the *Z. integrifolia* mitogenome against the plastid genome, retaining hits with e-value < 1e-5. Native mitochondrial homologues were excluded to avoid false positives, and overlapping hits were merged using BEDtools (Quinlan and Hall, 2010) to obtain non-redundant intervals. For each non-redundant fragment, the pairwise sequence identity to the extant plastome was calculated, and fragments were classified as recent (≥95%), intermediate (90 - 95%), or ancient (<90%). Plastid genes overlapping each MTPT were identified, and their full-length coverage and sequence integrity were assessed for embedded plastid genes.

### 5.5 Codon usage bias analysis

We evaluated codon usage patterns by analyzing the nucleotide composition and relative synonymous codon usage (RSCU) of the 40 identified PCGs. Calculations were performed in MEGA12 (Kumar et al., 2024) using the Standard Genetic Code. RSCU values—defined as the ratio of observed to expected codon frequency assuming equal usage of synonymous codons—were determined for each gene. Codons with RSCU > 1.0 were classified as preferred, while those < 1.0 were underrepresented. To assess mutational pressure effects, we calculated nucleotide frequencies at the first, second, and third codon positions (GC_1_, GC_2_, GC_3_) as well as at the whole-genome level.

### 5.6 RNA editing

C-to-U RNA editing sites were predicted using an ensemble of PlantC2U and MitoRED. Each cytosine in the mitochondrial protein-coding sequences was treated as a candidate site, and its 40-bp upstream and 40-bp downstream flanking regions were extracted to form an 81-bp context motif (40 bp + central C + 40 bp). MitoRED candidates with a probability ≥0.95 were retained and subsequently scored by PlantC2U. Final high-confidence editing sites were defined as the intersection of MitoRED (≥0.95) and PlantC2U (≥0.95) predictions. Codon position and synonymous/non-synonymous classification were determined from the corresponding CDS using the standard genetic code.

### 5.7 Phylogenetic analysis and substitution rates

We reconstructed the phylogenetic placement of *Z. integrifolia* using a dataset of mitochondrial genomes from 13 representative species and the angiosperm *Amborella trichopoda* (Rice et al., 2013) as outgroup. Ingroup sampling covered Cycadales: *Z. integrifolia*, *C. taitungensis* (Chaw et al., 2008); Ginkgoales: *G. biloba* (Guo et al., 2016); Pinaceae: *P. taeda* (NC_039746.1), *Abies koreana* (Park et al., 2024), *Larix sibirica* (Putintseva et al., 2020); Cupressaceae: *Platycladus orientalis* (Liu et al., 2022a), *Thuja sutchuenensis* (Xia et al., 2023), *Cunninghamia lanceolata* (Huang et al., 2026); Taxaceae: *Taxus chinensis* (OP177687), *Taxus wallichiana* (Qu et al., 2024), and Gnetales: *Welwitschia mirabilis* (Guo et al., 2016).

Mitochondrial protein-coding genes (PCGs) were utilized to infer phylogenetic relationships. Amino acid sequences for individual genes were aligned using MAFFT v7 (Katoh et al., 2002) and subsequently trimmed to remove poorly aligned and highly divergent regions. A custom Python script was used to filter genes based on sequence occupancy and abnormal tree lengths(present in at least 10 of the 13 taxa, total tree length < 2.5), and the retained alignments were concatenated into a supermatrix while generating a corresponding partition file. Phylogenetic analysis was performed using the Maximum Likelihood (ML) method implemented in IQ-TREE v2.2.0 (Nguyen et al., 2015). To account for rate heterogeneity across the mitogenome, ModelFinder (Kalyaanamoorthy et al., 2017) was employed to independently select the best-fit substitution model for each gene partition. Node support was evaluated with 1,000 ultrafast bootstrap replicates. In addition, a coalescent-based species tree was inferred with ASTRAL from the individual gene trees.

Because the Welwitschia branch was exceptionally long and could bias inference at deep nodes, all phylogenetic analyses (filtering, model selection, ML, gCF/sCF, and ASTRAL) were repeated after excluding Welwitschia as a sensitivity analysis for long-branch attraction. The final consensus tree was visualized and annotated using iTOL (Letunic and Bork, 2024).

### 5.8 Estimation of substitution rates and selective pressures

To evaluate the evolutionary dynamics and selective pressures acting on mitochondrial genes, we estimated the non-synonymous substitution rate (*dN*), synonymous substitution rate (*dS*), and their ratio (ω=*dN/dS*) for 35 conserved mitochondrial PCGs among *Z. integrifolia*, *C. taitungensis*, and the outgroup *G. biloba*. Codon-based alignments were generated, and lineage-specific evolutionary rates were estimated using the free-ratio branch model (model = 1) implemented in the codeml program of the PAML v4.10 package (Yang, 2007). To ensure statistical robustness, mathematical artifacts resulting from extreme evolutionary stasis or mutational saturation (e.g., dS<0.001 or artifactually inflated ω values) were excluded from downstream analyses. The statistical significance of differences in substitution rates and selective pressures among the distinct evolutionary lineages was assessed using a two-sided Mann-Whitney U test, followed by Bonferroni correction for multiple comparisons.

## Data Availability

The datasets presented in this study can be found in online repositories. The names of the repository/repositories and accession number(s) can be found below: https://www.ncbi.nlm.nih.gov/, PX874106.
